# A feasibility study: Using mobile phone-based tools to collect community-level Behavioral and Social Drivers (BeSD) of vaccination data in Zambia

**DOI:** 10.1371/journal.pgph.0004839

**Published:** 2025-09-16

**Authors:** Talya Shragai, Christina Riley, Mukuka Bwalya, Ester Sikare, Rose Tembo, Jacob Bibohere, Kalangwa Kalangwa, Winfridah Liwoyo Mulenga, Constance Simooya, Olivia Aguma, Kennedy Matanda, Mazyanga Mazaba Liwewe, Neetu Abad, Eugene Lam, Anna Winters, Kimberly E. Bonner

**Affiliations:** 1 Global Immunization Division, Centers for Disease Control and Prevention, Atlanta, Georgia, United States of America; 2 Akros, Lusaka, Zambia; 3 African Field Epidemiology Network, Kampala, Uganda; 4 Zambia Ministry of Health Promotion, Lusaka, Zambia; 5 Zambia Ministry of Health Expanded Program on Immunization, Lusaka, Zambia; 6 Zambia National Public Health Institute, Lusaka, Zambia; PLOS: Public Library of Science, UNITED STATES OF AMERICA

## Abstract

Routine collection of behavioral and social drivers (BeSD) of vaccination data is essential for understanding and addressing vaccine confidence and demand to achieve high vaccination coverage. Traditional house-to-house data collection methods are resource-intensive, prompting the need for alternative, scalable approaches. This study tested the feasibility of using mobile phone surveys to collect community-level BeSD data on COVID-19 vaccination in Zambia. A cross-sectional survey of adults aged 18 and over was conducted in three districts: Lusaka, Kalomo, and Chavuma. Participants were recruited via geotargeted mobile phone messages and responded using push-button inputs. The survey adapted validated BeSD questions from the World Health Organization (WHO) framework and was administered in English and six local languages. Strategies to increase response rates were tested, including offering a small monetary incentive and conducting community outreach via radio jingles. To assess the feasibility of using mobile phone surveys to collect BeSD data, we report on response rates and the demographic distribution of respondents and describe the operational process of applying this methodology. From March to July 2024, a total of 52,983 recruitment messages were sent, yielding an overall response rate of 15.7%. Response rates varied by district, with Chavuma having the highest (68.2%) and Lusaka the lowest (4.2%). Compared to a baseline response rate of 4.7%, offering a monetary incentive increased the response rate to 31.4%, while community outreach increased it to 19.8%. Respondents skewed younger (69.5% aged 18–29 years) and male (65.9%). Mobile phone surveys present a feasible method for collecting real-time BeSD data at the community level in low-resource settings. Incentives and community outreach effectively increase participation, though results may need to be weighted to reflect population demographics.

## Introduction

Vaccine hesitancy has been named as one of the top ten threats to global health by the World Health Organization (WHO) [1]. The COVID-19 pandemic led to substantial declines in childhood immunization coverage as well as decreased confidence in both COVID-19 vaccines and routine childhood immunizations [2,3]. In response, WHO has developed the Behavioral and Social Drivers (BeSD) of vaccination framework to better understand vaccine uptake and address barriers to vaccination [4]. These BeSD indicators provide a standardized, validated set of measures that enable countries to understand localized barriers to vaccination and develop targeted interventions to address them. If ministries of health can collect BeSD data routinely, it will provide even greater insight to understand how well interventions are working. This will allow for immunization programs to update and continuously improve implementation strategies for vaccine uptake.

WHO has expressed the need for regular collection, analysis, and use of BeSD of vaccination [4]. To this end, WHO has published standardized and validated surveys on BeSD for various vaccines, including one for COVID-19. These surveys were developed to increase the availability, standardization, and quality of data on the behavioral and social drivers of vaccination [4]. The WHO BeSD framework covers the full range of behavioral and social drivers of vaccination by assessing four domains that can affect vaccine demand and uptake: 1) thinking and feeling, 2) social processes, 3) motivation, and 4) practical issues [5,6]. These questions have been field-tested, validated, and applied in multiple countries [7,8].

However, few countries routinely collect BeSD data because the house-to-house data collection methodologies commonly applied to conduct BeSD surveys are costly, labor and time-intensive, and difficult to implement at the community level [9]. Other available methodologies, such as collecting BeSD data at health facilities, do not produce data that are representative of the general population [9]. There is a need to identify data collection methods for BeSD that are rapid, low cost, representative, and that can be routinely applied so that countries can easily collect and use high-quality data on the behavioral and social drivers of immunization to inform immunization programming decisions and targeted interventions.

With the expanded ownership of mobile phones and coverage of telecom service networks, mobile phone-based data collection methods have become a promising alternative to house-to-house surveys [10], and are already used for other health services such as immunization reminders [11,12]. Mobile phone ownership in low- and middle- income countries has doubled since 2008, and in Zambia, over 103 mobile phone subscriptions per 100 people were reported in 2020 [13]. Two common mobile phone-based survey methods are Short Message Service (SMS) and Interactive Voice Response (IVR) [10]. SMS and IVR-based surveys are remotely administered data collection methods that can rapidly reach populations with access to mobile phones and 2G coverage at low cost. SMS is best used for literate populations, while IVR can be applied in populations with a high proportion of low- or pre-literacy [14,15]. A benefit of these methods over other common mobile phone survey methods such as Computer-Assisted Telephone Interviewing (CATI) is that CATI requires staff to administer the interviews, while SMS and IVR surveys can be automatically administered with no staff required [10].

In our study, we piloted the feasibility of using SMS and IVR to collect community-level BeSD of COVID-19 data in Zambia. Zambia was selected because of its high mobile phone saturation and because the Zambia Ministry of Health is seeking to apply data-driven approaches to assess community knowledge and receive feedback on key behavioral barriers to uptake including myths about the COVID-19 vaccine, difficulty accessing vaccination sites, and vaccination stock-outs.

Our objective was to pilot and test the feasibility of mobile phone-based methods to collect data on validated COVID-19 BeSD core indicators at the community level using SMS and IVR. To assess feasibility, we report on the response rate to these surveys as well as factors affecting that response rate and the demographic distribution of respondents. We also report on the operational process to implement mobile phone surveys for BeSD data by providing a narrative description of the timeline, process, and successes/challenges of implementation.

## Methods

### Study design

This study used a cross-sectional design with BeSD of COVID-19 vaccine surveys distributed randomly to active mobile phone users of the Zamtel network in Lusaka, Chavuma, and Kalomo districts, Zambia. Surveys were self-administered through mobile phones, with participants receiving an initial recruitment message to participate using either SMS or IVR and opting into and responding to multiple choice survey questions via push button. Computer-Assisted Telephone Interviewing (CATI) was not tested here, because while this methodology is widely used for health surveys and there is evidence that this methodology produces higher response rates [16,17], we were interested in testing methods that require minimal human labor and cost. We tested the impact of several survey strategies on response rate, including 1) the time of day the recruitment message was sent, 2) whether the survey was sent in a single language or multiple languages, 3) which of two versions of the recruitment message was received (a ‘call to action’ vs. no ‘call to action,’ see S1 Text), and 4) if the participant was offered a small incentive in the recruitment message to be paid upon completing the survey. A factorial design was used to test the marginal effects of these strategies on response rates. We also tested the response rate before and after community outreach activities, which consisted of radio jingles encouraging the respondent to opt-in to the survey. The sequencing of how these strategies were implemented and tested is described below. Survey questions were adapted for mobile phones from the WHO-validated set of BeSD of COVID-19 questions and included basic respondent demographic questions (S1 Text).

### Setting and participants

The three intervention districts (Lusaka, Chavuma, and Kalomo) were selected because 1) phone numbers were available to investigators and these districts had high cellular network coverage; 2) the COVID-19 vaccine had been rolled out to the general population of adults ≥18 years old for a minimum of one year; and 3) they represented one rural, one peri-urban, and one urban district. These districts were selected in consultation with the Ministry of Health, Expanded Program on Immunization, Health Promotion Bureau, and the Zambia National Public Health Institute. Additional selection criteria that were considered included smartphone versus basic mobile phone saturation, literacy rates, district COVID-19 vaccination uptake rates, urbanicity and access to the rail line, other sociodemographic factors, and whether the district was Gavi-supported (indicating programmatic support for increased vaccination).

The study population included eligible individuals who opted into the survey via short-code response across each of the three districts with the goal of capturing a random subset of the general adult population. Participants were considered eligible if they 1) resided in the selected study areas, 2) were 18 years or older, and 3) gave informed consent. Eligibility was determined by participant self-report through push button response before the survey was shared.

### Operational process

To implement the two-way messaging system for collecting BeSD survey data, we first registered and licensed a short code with the Zambia Information and Communications Technology Authority (ZICTA). This short code allowed participants to engage with the survey via SMS or IVR. Next, we contracted with local telecommunications providers to obtain active anonymous phone number lists for our sampling frame and to enable geographic targeting of recruitment messages based on subscriber location data. Contracts were pursued with three telecommunications providers—Zamtel, Airtel, and MTN—each covering over 37% of Zambia’s mobile network by population.

We then partnered with a third-party mobile survey provider, Ontech Solutions, to configure the messaging system. This involved developing and testing two-way SMS and IVR messaging on the systems and creating a data management pipeline to collect and transfer responses for analysis. To incentivize participation, we integrated a mobile money payment system that transferred five kwacha (approximately $0.20 USD) to respondents upon survey completion. The monetary incentive was only offered during the phase of the project when the incentive strategy was being tested. An anti-fraud system was also developed to ensure that each phone number could only participate once and receive a single incentive.

To assess the effect of community outreach on survey response rates, we worked closely with the Health Promotion team at the Zambia Ministry of Health to develop two radio jingles. These jingles were created in consultation with community focus groups and refined based on participant feedback. Radio jingles provided listeners with the short code to opt-in and participate via SMS or IVR and were broadcast on local radio stations in Lusaka (HOT FM, Power Radio, Christian Voice Radio) and Kalomo (Voice of Kalomo Community Radio). Radio jingles were aired in English and the most common local language for that jurisdiction. Since Chavuma lacked a local radio station, we aired the jingles on Zambia’s National Broadcaster (ZNBC) in the local language of Chavuma, Luvale, one week after broadcasts in Lusaka and Kalomo.

**Fig 1:**
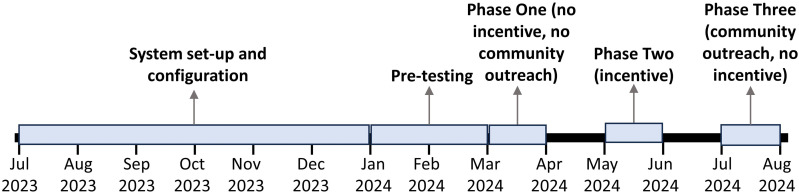
Timeline of pilot from July 2023 to July 2024. Timeline includes system set-up and configuration, pre-testing and refinement of the messaging system, and phase one (messaging without incentives or community outreach), phase two (messaging with the provision of a small monetary incentive on completion of the survey), and phase three (messaging with the concurrent airing of radio jingles as community outreach). Passive recruitment was available throughout the entire testing period.

The overall timeline for the project activities is shown in Fig 1. The messaging component was implemented during all three phases, with variation in time of day sent, language used, and type of recruitment message sent.

### Data collection & management

Based on existing literature on maximizing quality and completion of mobile phone surveys, the survey was limited to 13 questions. The survey included nine priority questions from each category of the BeSD question bank as well as participant age, current participant district of residence, sex, and if they provide informed consent and met study criteria. Those who responded that they did not meet the study criteria or did not provide informed consent were not given access to the survey. Questions selected were adapted for mobile phone to meet character limits. The survey was translated into 6 local languages (Bemba, Nyanja, Tonga, Luvale, Lunda, Kaonde), with all translations approved by both the Institutional Review Board ERES of Zambia and the Zambia National Health Research Authority. The full survey with questions and response options in English is provided in S1 Text. Surveys were tested on both smart and non-smart phones to ensure compatibility with all phone types.

Recruitment messages were sent to randomly selected phone numbers in each of the three districts selected by the MOH using the Zamtel active number list. Phone numbers were randomly selected from the list per district using a random number generator. Surveys were geo-targeted to study districts based on Zamtel data on the closest cell tower where mobile phones had been most recently active. The recruitment message was sent by bulk SMS delivery; recipients could opt in to participate to the survey via the provided short code. Participants answered survey questions using push button based on multiple choice options for each question. The text of all recruitment messages used is included in S1 Text.

The surveys were divided into three data collection phases of approximately three weeks each. The Phase One ‘baseline’ phase was conducted without providing any monetary incentive for participation and with no community outreach. In Phase Two, half of the participants were randomly selected to be offered in the recruitment message a small incentive of five kwacha (about $0.20 USD) in the form of mobile money to be paid upon completion of the survey. In Phase Three, we concurrently ran community outreach radio jingles while sending recruitment messages. No incentive was offered during the community outreach phase.

Several messaging strategies were tested during recruitment that were hypothesized to have an effect on response rate: 1) Whether the survey modality was SMS versus IVR 2) the time of day the recruitment message was sent (either 11am or 5pm); 3) whether the survey recruitment message was sent once in the most common language spoken in the targeted district or twice in the two most commonly spoken language of each district; 4) and which of two developed versions of the recruitment message was received, ‘call to action’ or no ‘call to action’ (see text in S1 Text). A factorial design for messages sent was used in order to be able to test the marginal effects of these strategies (time of day, language strategy, and version of the recruitment message), with the combination of survey strategies randomly and equally distributed among messages sent. Only main-effect comparisons were analyzed; no interaction effects were tested.

All data received were stored on a secured cloud-based servers at Ontech Solutions. Data were shared from the Ontech platform to the Akros platform via an API. Cleaned data for analysis were shared with CDC via a secure data sharing site (ShareFile). All messages were end-to-end encrypted, and all data transfer and sharing were also encrypted. Data from all survey responses from eligible participants, including those who did not complete the full survey, were included in the analysis. Data analysis was completed in Stata [18] and R (4.41).

### Data analysis

We first analyzed overall response rate and then disaggregated it by district and by survey phase (baseline, monetary incentive, and community outreach). Response rates are defined here using the standard American Association for Public Opinion Research (AAPOR) Response Rate 2 definition, which is calculated by dividing the number of complete and partial interviews by the total number of interviews (complete plus partial), plus the number of non-interviews (refusals, break-offs, non-contacts, etc.), plus any cases of unknown eligibility [18]. In other words, a response was defined as receiving at least one response to a survey question and response rate was calculated as the number of people who sent in at least one response divided by the number of recruitment messages sent. Response rate was compared by district and by survey phase using Chi-squared tests.

In addition to responses from phone numbers that received a survey recruitment message (active recruitment), we also received responses from phone numbers that did not receive a recruitment message (passive recruitment). These passive recruitment responses are presumably from individuals who responded to a community outreach radio ad or saw a friend or family member receive a recruitment message. We compare the number of responses per survey phase that were received via active and passive recruitment using Chi-squared tests.

To calculate response rate by messaging strategy, we restricted data to phone numbers that received a recruitment message (active recruitment). Response rate was compared by messaging strategy using Chi-squared tests.

Next, we calculated the completion rates of the survey among respondents, defined as the percent of people who completed all survey questions, and compared completion rate between those who were offered a mobile money incentive for completing the survey and those who were not using Chi-squared tests.

Finally, we compared the demographics of survey respondents by sex, age group, and if the respondent reported being vaccinated against COVID-19 using Chi-squared tests. Age was categorized into three groups: 18–29, 30–49, and 50+. Self-reported sex, age, and vaccination status were used for these analyses.

We use a significance threshold of 0.05 for all statistical tests. Respondents less than 18 years old were excluded from analysis.

The distribution of responses to the survey itself are included as supplementary material in S1 Table.

### Ethical considerations

This study was reviewed and approved by the Institutional Review Board ERES of Zambia and the Zambia National Health Research Authority. This activity was reviewed by CDC, deemed not research, and was conducted consistent with applicable federal law and CDC policy (§ See, e.g., 45 C.F.R. part 46, 21 C.F.R. part 56; 42 U.S.C. §241(d); 5 U.S.C. §552a; 44 U.S.C. §3501 et seq.). The findings and conclusions in this report are those of the authors and do not necessarily represent the official position of the Centers for Disease Control and Prevention.

### Patient and public involvement

The public were not involved in the design, or conduct, or reporting, or dissemination plans of our research. Community feedback on the radio jingles was taken into consideration.

## Results

### Operational process

#### Timeline.

The operational process for conducting the mobile phone survey began with the registration and licensure of the short code by ZICTA, which was completed within one month. Contracting and configuration with the selected third-party survey provider, Ontech, was finalized by July 2023 after three months. Of the three telecom companies pursued, only Zamtel was fully successful. Zamtel’s contracting and configuration took approximately six months and included the provision of an anonymized active number list for each of the three study districts in order to conduct geotargeted recruitment. Contracting and configuration with Airtel was completed after one year but did not allow for geotargeting of Airtel subscribers. Efforts to contract with MTN were abandoned after eight months due to technical and contractual limitations. System testing and pre-testing phases were conducted from August to December 2023 and January to February 2024, respectively, followed by full data collection from March to July 2024.

#### Challenges and successes.

One of the key successes of the operational process was contracting with Zamtel, which provided a geographically targeted active phone number list. This list was based on the cell phone tower to which subscribers were closest for the greatest number of days over the last 90 days, allowing for targeted recruitment in the three selected districts. Because Airtel was not able to provide geotargeted messaging this limited our ability to recruit Airtel subscribers, and we were not able to reach MTN subscribers. These limitations reduced the overall reach of the survey, particularly in districts where Zamtel’s market share was lower.

While Zamtel provided geolocation metadata, inconsistencies were observed between telecom-provided locations and participant-reported locations. For example, while 4,340 total respondents were listed as located in Chavuma by the Zamtel active number list, only 53% of these self-reported as being located in Chavuma at the time of the survey; the percent agreement between the active numbers list and self-report for Kalomo and Lusaka was higher at 85% and 96%, respectively. This discrepancy highlighted a need for better alignment between telecom data and participant-reported demographics in future iterations.

### Survey response rates

**Fig 2:**
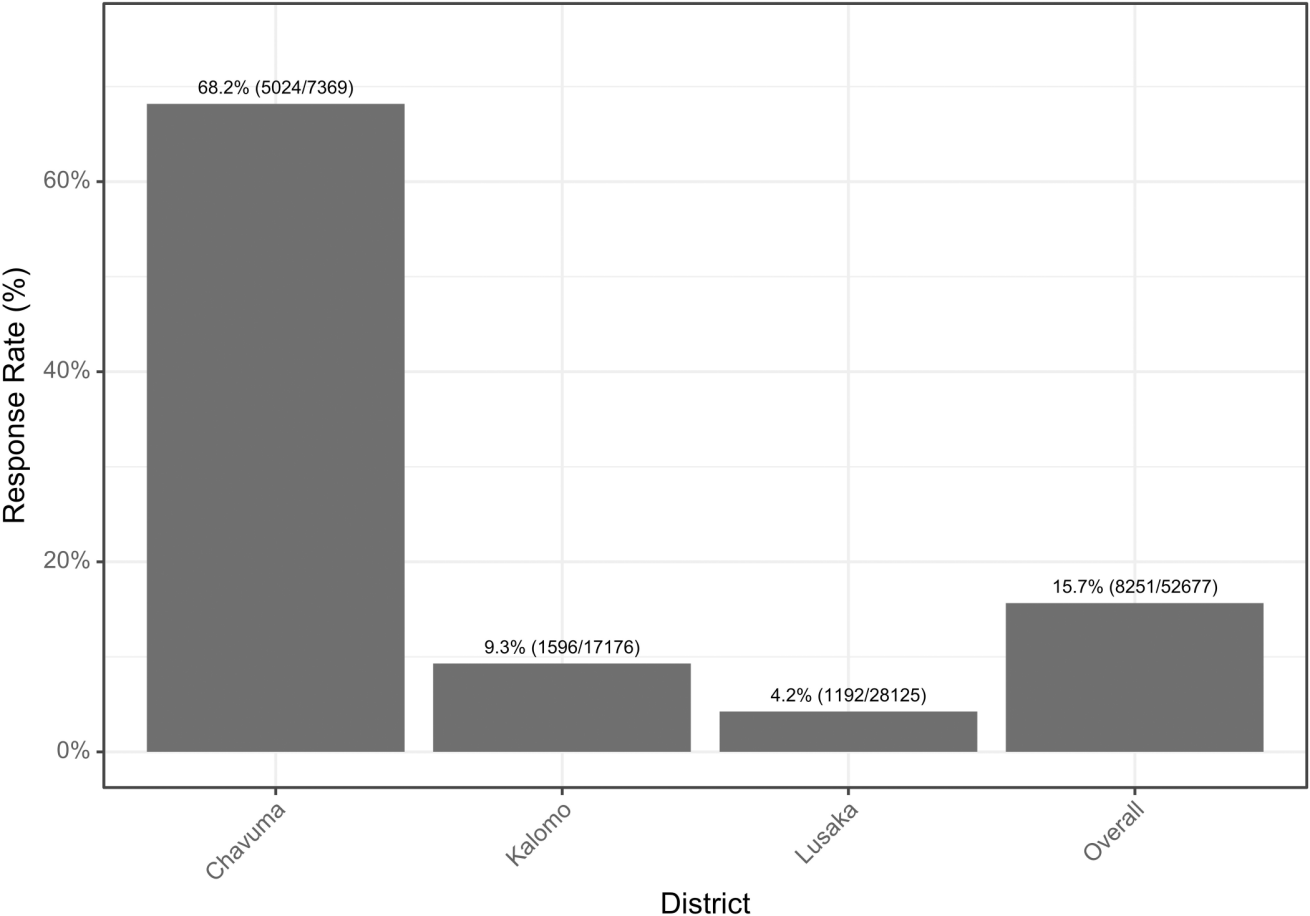
SMS/IVR survey response rate by district. Response rate is calculated as all responses, both through active and passive recruitment, divided by the total number of recruitment messages sent across all phases.

Over the data collection period between March and July 2024, a total of 52,677 recruitment messages were sent, with 7,369 sent to Chavuma, 17,176 to Kalomo, and 28,125 to Lusaka. Overall 8,251 responses were received, yielding a response rate of 15.7%. Response rate was highest in Chavuma at 68.2% (5024/7369), second highest in Kalomo at 9.3% (1596/17,176), and lowest in Lusaka at 4.2% (1192/28,125) (Fig 2). Response rate in Chavuma was higher than in Lusaka (X-squared = 16525, P < 0.001) and higher in Kalomo than in Lusaka (X-squared = 471.57, df = 1, P < 0.001).

**Fig 3:**
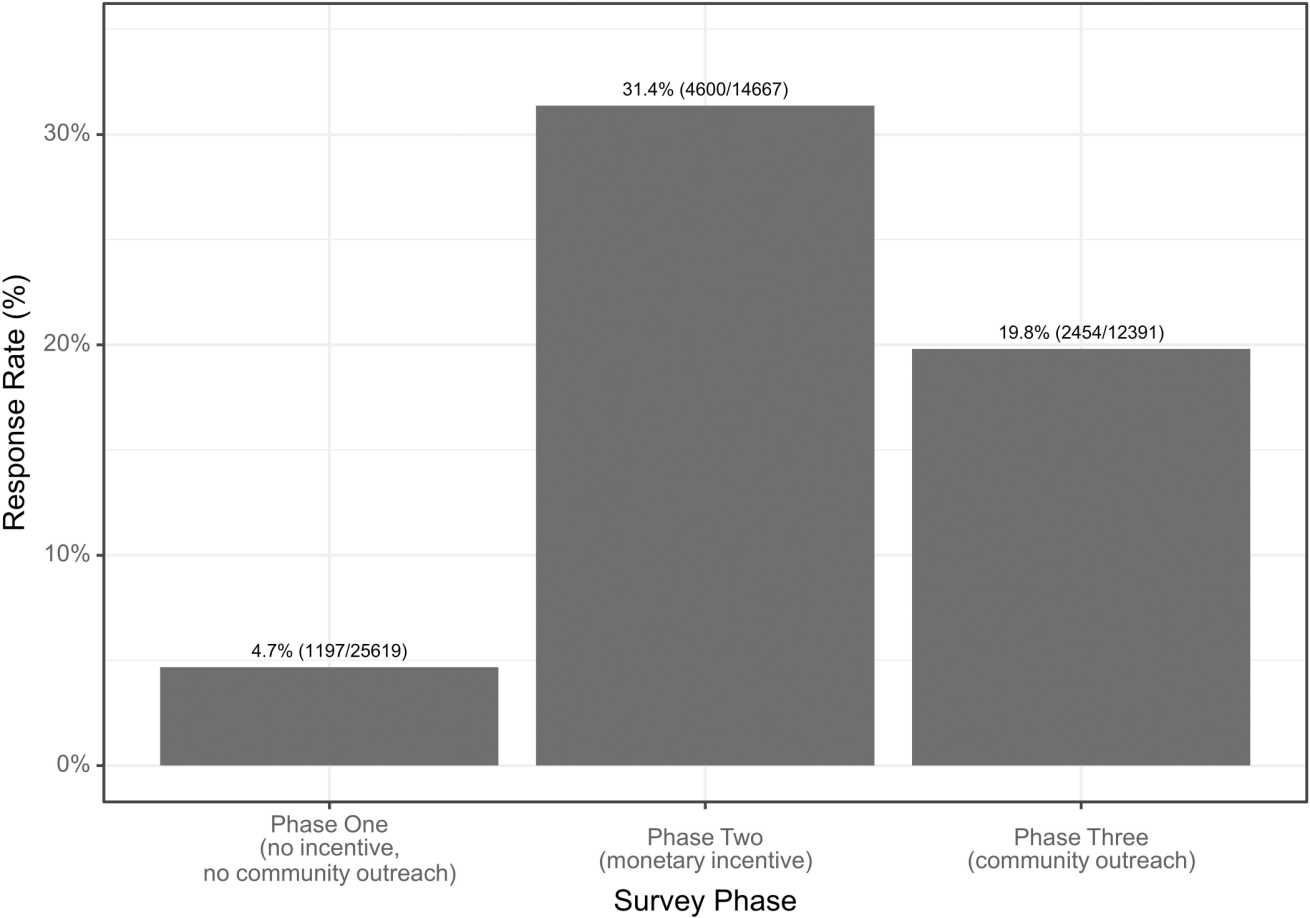
Response rate per survey phase. Survey phases include Phase One (no incentive or community outreach), Phase Two (a small monetary incentive offered on completion of the survey), and Phase Three (community outreach by radio jingles concurrent with recruitment messages).

Response rate varied by survey phase. Response rate for Phase One at baseline (without mobile money incentives and before community outreach) was 4.7%, response rate in Phase Two when mobile money incentives were included was 31.4%, and response rate in Phase Three after community outreach radio jingles was 19.8% (Fig 3). Response rate in Phase Two was higher than in Phase One (X-squared = 5393.7, df = 1, P-value <0.001) and response rate in Phase Three was also higher than in Phase One (X-squared = 2202.6, df = 1, P-value <0.001).

The proportion of active (participants who received a recruitment message) versus passive (participants who sent in a response but did not receive a recruitment message) responses was 51.7% active and 48.3% passive in Phase One without mobile money incentives and before community outreach. This proportion was similar in Phase Two, compared to Phase One, when participants received a mobile money incentive (49.4% active, 50.6% passive, X-squared = 1.88, df = 1, P-value = 0.17), but a higher proportion were passive in Phase Three, compared to Phase One, when community outreach was conducted (20.7% active, 79.3% passive, X-squared = 360, df = 1, P-value<0.001).

Response rate among those who received a recruitment message was similar by SMS vs IVR modality (3.5% vs 3.3%, (X-squared = 1.367, df = 1, P-value = 0.24)). Response rate was significantly higher when the message was sent in the morning (11am) vs in the evening (5pm) (3.9% vs 2.4%, (X-squared = 70.06, df = 1, P-value<0.001)). No significant different in response rates was detected between the two wordings of the recruitment message (3.5% for no call to action vs 3.3% for using a call to action overall (X-squared = 1.03, df = 1, P-value = 0.31)), and when the recruitment message was sent in the most commonly spoken language per district vs multiple times in multiple languages (3.4% for multiple messages in multiple languages vs 3.4% for one time in the most commonly spoken language per district (X-squared = 0.08, df = 1, P-value = 0.78)).

Among respondents who initiated the survey, completion rate was 74% when a mobile money incentive was not offered and was higher at 86% when a mobile money incentive was offered (X-squared = 215.06, df = 1, P-value<0.001).

Overall, 9.0% of respondents reported that they were unvaccinated against COVID-19 while 75.7% reported that they had received a COVID-19 vaccine. Respondents were skewed by age (X-squared = 13056, df = 4, P-value<0.001) and sex (X-squared = 4131.8, df = 2, P-value<0.001), with the majority between 18–29 (74.3%) and male (65.9%). These results are summarized in Table 1.

**Table 1:** Distribution of respondents by sex, age group, and COVID-19 vaccination status. All respondent demographics are self-reported.

	Respondents, N = 8251, n (%)
**Sex**	
Male	5437 (65.9%)
Female	1925 (23.3%)
Unknown (no response)	889 (10.1%)
**Age group**	
<18 (ineligible, excluded from analysis)	314 (3.8%)
Age 18–29	5738 (69.5%)
Age 30–49	1317 (16.0%)
Age 50+	355 (4.3%)
Unknown (no response)	527 (6.4%)
**Vaccination status**	
Vaccinated	6248 (75.7%)
Not vaccinated	739 (9.0%)
Unknown (no response)	1264 (15.3%)

## Discussion

This study piloted mobile phone-based tools to collect community-level BeSD data in three districts in Zambia. Our primary objective was to evaluate the feasibility of conducting these mobile phone surveys, focusing on operational processes, factors influencing the response rates, and the demographic distribution of respondents. Additionally, the findings provide insights into the behavioral and social drivers of COVID-19 vaccination in Zambia. Overall, this study demonstrated that mobile phone-based surveys can be used to capture vaccine demand data.

The overall response rate in this study was 15.7%, with substantial variation between districts. Chavuma, a rural district, had a significantly higher response rate (68.1%) compared to the urban district of Lusaka (4.2%). This may be because those in Chavuma were potentially more willing to participate in mobile phone surveys, or it may be because Zamtel is a less popular telecom provider in both Lusaka and Kalomo. As supported by the literature and anecdotal evidence from discussion at the dissemination meeting, residents in more urban areas receive a high level of spam SMS messages on a regular basis [19], which may have also decreased the response rate in Lusaka and Kalomo. Both overall response rate and completion rates align with similar studies using mobile surveys for public health in low- and middle-income countries [20,21]. Offering a small mobile money incentive in the initial recruitment message to be paid on completion of the survey significantly boosted response rates (31.4% with incentives vs. 4.7% without) and increased the survey completion rate (86% with incentives vs. 74% without). These findings suggest that small monetary incentives can be a useful tool for improving data quality and participation in mobile phone-based surveys. Future users will need to weigh the benefits against the financial sustainability of doing so in larger programs. Community outreach via targeted radio jingles also increased response rates, which rose to 19.8% post-outreach compared to 4.7% before. Notably, a large proportion of these responses were from phone numbers that did not receive a recruitment message, likely coming from participants who responded to the radio jingles, indicating that this strategy might be an effective supplemental method to collect BeSD data from the community and could potentially be an important strategy for future recruitment. However, the geographic limitations of radio coverage, especially in areas without local stations, present challenges for scaling community outreach nationwide or granular geotargeting of these surveys.

Respondents utilized both SMS and IVR modalities, and preference per modality varied by district. IVR performed better in Chavuma (41.3% response rate) compared to SMS (26.0%), while in Kalomo, SMS had a higher response rate (3.9% for SMS vs. 1.2% for IVR). Offering both SMS and IVR modalities is essential to ensure equitable access to BeSD surveys, particularly for pre-literate populations [21]. Of the strategies tested, sending recruitment messages in the morning led to slightly better response rates than sending them in the evening (11 am vs. 5 pm). Other variables, such as message language and wording, had minimal impact on response rates. These factors may vary in different contexts, and future studies should test and refine recruitment strategies.

Demographically, the survey skewed towards younger (18–29 years) and male respondents. This bias likely reflects mobile phone ownership trends in Zambia, which also skew heavily toward a young and male population [16]; furthermore these results are not outside the norm of what is expected for mobile phone surveys [22]. Additionally, a larger proportion of respondents reported being vaccinated than the reported vaccination coverage rate in Zambia (>70% nationally), indicating that those with more positive health behaviors were more likely to participate. These biases have been documented in other studies, summarized in [21], and may be particularly important to address for childhood vaccination surveys where females are more likely to be the decision-makers around vaccination. As has been suggested in other literature [22], this suggests that future surveys will need to weigh results to be able to use these data for programmatic decision-making and that strategies should be explored to target older, female demographics.

The study had several operational challenges. While we were able to contract with telecoms in Zambia with the support of the Ministry of Health, we were not able to contract with all three major telecommunication companies. Contracting with multiple telecom companies is critical, particularly because usage between companies varies widely by district in Zambia. Discrepancies between telecom metadata and respondents’ reported locations, particularly in Chavuma, highlighted the need for more reliable geographic targeting. Closer collaboration with telecom companies or the exploration of alternative targeting methods, such as broadcasting messages via cell towers, may enhance the accuracy of future recruitment efforts. Transitioning this study to an established, Ministry of Health-owned system may mitigate some of the issues experienced in coordinating with telecom companies during this pilot. In other countries, the ability to develop contracts between Ministries of Health and telecoms companies may be context dependent and based on existing relationships between telecoms and government.

This study had several key limitations and important considerations that should be applied to future use. First, we were unable to correct our analysis for the 60% of recruitment messages that were sent but not received, which significantly impacted the response rate. We also lacked demographic information on the sampling frame of phone numbers, limiting our analyses. Future work with telecom companies should ensure that a line-listed report of messages is successfully received and more demographic information of the line list can be obtained. Additionally, relying on a single telecom provider restricted the survey’s reach, particularly in districts where Zamtel is not the preferred provider. Expanding to multiple telecom providers in future studies could improve coverage. This study also did not verify the self-reported responses received by the mobile phone survey and all responses were based on participant recall, nor did it directly compare results to a more established methodology; for example, a proportion of unvaccinated respondents reported that they had received a booster, which may indicate some issues with data validity or contextual understanding of the survey options. While there is some evidence that IVR surveys are not inferior to CATI surveys for health [23], additional future studies should explore how the results of a BeSD survey conducted by mobile phone directly compare to other well-established methodologies such as a house-to-house survey. Finally, the focus on COVID-19 vaccination targets all adults over 18 years, while many other vaccination surveys target a much smaller population, such as caretakers of children under two. Future studies are needed to test the effectiveness of this methodology for routine immunization BeSD surveys where the demographic of respondents is focused on a narrower population.

Despite these limitations, this pilot demonstrates that mobile phone-based surveys are a promising method for collecting BeSD data that can be used for programmatic decision making. Based on the results of this study, the Ministry of Health of Zambia is interested in scaling up this system for further use. Lessons learned from this study can be applied to routine immunization and inform broader-scaled BeSD data collection both in Zambia and other lower-middle income countries. While challenges remain and further refinement will be necessary, mobile-based surveys offer an alternative for collecting real-time vaccine demand and attiute data.

## Supporting information

S1 Table Responses to Behavioral and Social Drivers (BeSD) survey questions stratified by vaccination status. Unknown responses are excluded from table and from percentage calculations.
(DOCX)

S1 Text Full recruitment message and survey text, including response options to each survey question. (DOCX)
