# Supplementary material for: A feasibility study: Using mobile phone-based tools to collect community-level Behavioral and Social Drivers (BeSD) of vaccination data in Zambia
Source: PLOS Glob Public Health. 2025 Sep 16;5(9):e0004839. doi: 10.1371/journal.pgph.0004839 (PMC12440194; doi:10.1371/journal.pgph.0004839)
Supplement: S1 Table — Unknown responses are excluded from table and from percentage calculations. (DOCX) [file pgph.0004839.s001.docx]

**S1 Table.** Responses to BeSD survey questions stratified by vaccination status. Unknown responses are excluded from table and from percentage calculations.

|  | **Unvaccinated N=739, n (%)** | **Vaccinated N=6248, n (%)** |
| --- | --- | --- |
| **How concerned are you about COVID-19?** |  |  |
| Not at all | 82 (11.5%) | 131 (2.1%) |
| A little | 38 (5.3%) | 66 (1.1%) |
| Moderately | 105 (14.7%) | 171 (2.8%) |
| Very | 490 (68.5%) | 5839 (94.1%) |
| **How important do you think COVID-19 vaccines are?** |  |  |
| Not at all | 73 (10.5%) | 82 (1.3%) |
| A little | 15 (2.2%) | 30 (1.3%) |
| Moderately | 77 (11.1%) | 136 (2.2%) |
| Very | 531 (76.3%) | 5868 (96.0%) |
| **Have most family and friends received COVID-19 vaccine?** |  |  |
| Yes | 349 (49.9%) | 5716 (93.2%) |
| No | 239 (34.2%) | 195 (3.2%) |
| Don’t know | 111 (15.9%) | 225 (3.7%) |
| **Do you think most close family and friends want you to get vaccinated against COVID-19?** |  |  |
| Yes | 483 (70.0%) | 5648 (92.6%) |
| No | 126 (18.3%) | 191 (3.1%) |
| Don’t know | 81 (11.7%) | 260 (4.3%) |
| **Do you know where to get a COVID-19 vaccine for yourself or where to refer anyone interested?** |  |  |
| Yes | 335 (49.3%) | 5623 (92.5%) |
| No | 345 (50.7%) | 453 (7.5%) |
| **Do you find going to get a COVID-19 vaccine costly?** |  |  |
| Not at all | 102 (15.2%) | 1083 (17.9%) |
| A little | 27 (4.0%) | 340 (3.8%) |
| Moderately | 103 (15.4%) | 533 (8.8%) |
| Very | 437 (65.3%) | 4181 (69.3%) |
| **Do you want to get a COVID-19 booster vaccine?** |  |  |
| Yes, have already received | 253 (38.6%) | 4922 (82.0%) |
| Yes, want to | 296 (45.1%) | 886 (14.8%) |
| Not sure | 43 (6.6%) | 85 (1.4%) |
| Do not want | 64 (9.8%) | 111 (1.8%) |
