# Supplementary material for: A feasibility study: Using mobile phone-based tools to collect community-level Behavioral and Social Drivers (BeSD) of vaccination data in Zambia
Source: PLOS Glob Public Health. 2025 Sep 16;5(9):e0004839. doi: 10.1371/journal.pgph.0004839 (PMC12440194; doi:10.1371/journal.pgph.0004839)
Supplement: S1 Text — (DOCX) [file pgph.0004839.s002.docx]

**S1 Text.** Full recruitment message and survey text, including response options to each survey question.

Recruitment message text A (non call-to-action):

1. IVR: Zambia MOH is conducting a survey; you will NOT be charged airtime to participate. Please dial 4300 to participate.
2. SMS: Zambia MOH is conducting a survey; you will NOT be charged airtime to participate. Please text YES to 4300 to participate.

Recruitment message text B (call-to-action):

1. IVR: Respond to this FREE MOH survey to help protect your community from COVID-19. Please dial 4300 to participate.
2. SMS: Respond to this FREE MOH survey to help protect your community from COVID-19. Please text YES to 4300 to participate.

Initial text:

This message is from researchers at MOH, ZNPHI, Akros, AFENET, and the US CDC. Are you 18 or older, and do we have your consent for this survey? Text YES if you give your consent AND are 18 or older, and NO otherwise.

MOH & Akros are conducting a survey:

**Question 1: Language Selection**
Please select your language:
a) English
b) Nyanja
c) Bemba
d) Tonga
e) Kaonde
f) Lunda
g) Luvale

**Question 2: What is your age?**
(Enter your age in years)

**Question 3: What is your gender?**
a) Male
b) Female

**Question 4: In which district do you currently reside?**
a) Lusaka
b) Chavuma
c) Kalomo

**Question 5: In which compound or community do you currently reside?**
(Select from dropdown)

**Question 6: Have you received a COVID-19 vaccine?**
a) Yes
b) No

*Skip Pattern: If “No” for Question 6, proceed to Question 7. Otherwise, skip to Question 8.*

**Question 7: Do you want to get a COVID-19 vaccine?**
a) Yes, I do want to
b) No, I do not want to
c) Not sure

**Question 8: How concerned are you about getting COVID-19?**
(Rank from 1 [Very Concerned] to 4 [Not at all concerned])
a) Very Concerned
b) Somewhat concerned
c) A little concerned
d) Not at all concerned

**Question 9: How important is getting a COVID-19 vaccine for your health?**
a) Very important
b) Somewhat important
c) A little important
d) Not at all important

**Question 10: Have most of your close family and friends received the COVID-19 vaccine?**
a) Yes
b) No
c) Don’t know

**Question 11: Do you think most of your close family and friends want you to get a COVID-19 vaccine?**
a) Yes
b) No
c) Don’t know

**Question 12: Do you know where to get a COVID-19 vaccine for yourself?**
a) Yes
b) No

**Question 13: Do you find it costly to get a vaccine?**
(Consider clinic costs, transport, or missed work)
a) Very costly
b) Somewhat costly
c) A little costly
d) Not at all costly

**Question 14: Do you want to get a COVID-19 booster vaccine?**
a) Yes, I have already received a COVID-19 booster
b) Yes, I do want to
c) Not sure
d) No, I do not want to
